# Functional Genomics of the *Aeromonas salmonicida* Lipopolysaccharide O-Antigen and A-Layer from Typical and Atypical Strains

**DOI:** 10.3390/md13063791

**Published:** 2015-06-15

**Authors:** Susana Merino, Elena de Mendoza, Rocío Canals, Juan M. Tomás

**Affiliations:** 1 Department of Microbiology, Faculty of Biology, University of Barcelona, Diagonal 643, Barcelona 08071, Spain; E-Mails: elenademendoza@hotmail.com (E.M.); jtomas@ub.edu (J.M.T.); 2 Institute of Integrative Biology, University of Liverpool, Crown Street, Liverpool L69 7ZB, UK; E-Mail: rcanals@gmail.com

**Keywords:** *Aeromonas salmonicida*, subspecies, genomics, proteomics, lipopolysaccharide O-antigen, A-surface layer

## Abstract

The *A. salmonicida* A450 LPS O-antigen, encoded by the *wb*_salmo_ gene cluster, is exported through an ABC-2 transporter-dependent pathway. It represents the first example of an O-antigen LPS polysaccharide with three different monosaccharides in their repeating unit assembled by this pathway. Until now, only repeating units with one or two different monosaccharides have been described. Functional genomic analysis of this *wb*_salmo_ region is mostly in agreement with the LPS O-antigen structure of acetylated l-rhamnose (Rha), d-glucose (Glc), and 2-amino-2-deoxy-d-mannose (ManN). Between genes of the *wb*_salmo_ we found the genes responsible for the biosynthesis and assembly of the S-layer (named A-layer in these strains). Through comparative genomic analysis and in-frame deletions of some of the genes, we concluded that all the *A. salmonicida* typical and atypical strains, other than *A. salmonicida* subsp. *pectinolytica* strains, shared the same *wb*_salmo_ and presence of A-layer. *A. salmonicida* subsp. *pectinolytica* strains lack *wb*_salmo_ and A-layer, two major virulence factors, and this could be the reason they are the only ones not found as fish pathogens.

## 1. Introduction

Currently, there are five accepted subspecies of *Aeromonas salmonicida*: *A. salmonicida* subsp. *salmonicida*, *masoucida*, *achromogenes*, *pectinolytica* and *smithia* [1]. *Aeromonas salmonicida* subsp. *salmonicida* is the etiological agent producing the systemic disease named furunculosis, being then an important fish pathogen [2]. This pathogen has been subjected to considerable investigation due to its importance in the farmed fish industry. Its major virulence factor is an S-layer (named A-layer), which principally consists of a unique two-dimensional crystalline tetragonal protein (A-protein with a molecular weight of 49 kDa) array [3], which is tethered to the cell by the lipopolysaccharide (LPS) [4].

Immunolabeling studies have been able to show that the A-layer appears to cover most of the surface of the virulent *A. salmonicida* [5], nevertheless some LPS molecules are still surface exposed [6]. Both LPS O-antigen and the A-layer are required to fully protect this bacterium from serum killing [7]. Although the A-layer is not completely necessary for the bacterium’s resistance to serum killing, it is an important barrier against opsonophagocytosis [8].

In Gram-negative bacteria, the LPS are large amphiphilic molecules consisting of a hydrophilic polysaccharide part, and a covalently bound hydrophobic and highly conserved lipid component, termed lipid A (the bioactive endotoxin subunit). The polysaccharide part can be conceptually divided into two sub-domains: one more internal and conserved, the core region, and one more external and highly variable, the O-specific chain, named also O-antigen for its immunogenic properties. These three regions have been differentiated and formally classified by their chemical structure, degree of conservation, biosynthetic pathways and genetic determination (see general review, [9]).

Some studies have chemically characterized structures of the O-antigen polysaccharide and the core oligosaccharide regions of the LPS from *A. salmonicida* strain SJ-15 [10,11]. More recent studies describe the structural elucidation of the O-antigen LPS of the *A. salmonicida* subsp. *salmonicida* from strains A449 and 80204-1 [12], and their core oligosaccharide region [13]. We studied the functional genetics of the O-antigen of the LPS from *A. salmonicida* subsp. *salmonicida* strain A450, whose chemical structure is similar to the previously described for other strains [12]. Furthermore, we found genes encoding for the production and export/assembly of the A-layer characteristic from *A. salmonicida* subsp. *salmonicida* strains, between the biosynthetic genes for the LPS O-antigen production (cluster named *wb*_salmo_). We also studied the *wb*_salmo_ and genes encoding for the production and export/assembly of the A-layer in different strains of subspecies *masoucida*, *achromogenes*, *pectinolytica* and *smithia*.

## 2. Results

LPS was extracted from enzymatically digested *A. salmonicida* A450 cells by the Westphal procedure [14] and the O-polysaccharide isolated after mild acid degradation. Sugar analysis by gas-liquid chromatography (GLC) of resultant monosaccharides as alditol acetates and (*S*)-octyl glycosides revealed that it was composed of l-rhamnose (Rha), d-glucose (Glc), and 2-amino-2-deoxy-d-mannose (ManN) in the approximate molar ratio of 1:0.95:0.9. The methylation analysis of the *O*-polysaccharide revealed the presence of 2,3-di-*O*-methylrhamnose, 2-*O*-methylrhamnose, 2-deoxy-4,6-di-*O*-methyl-2-(*N*-methylacetamido) mannose and 2,3,4,6-tetra-*O*-methylglucose (Table 1), suggesting that the O-antigen polysaccharide contained 3,4-substituted Rha, 3-substituted ManNAc and terminal Glc.

**Table 1:** *A. salmonicida* A450 O-antigen LPS methylation analysis.

Sugar Linkage	Rt_GM_^a^ (min)	Relative Molar Ratios ^b^
4-Substituted Rha	5.19	0.07
Terminal Glc	6.18	1.00
3,4-Substituted Rha	7.03	0.85
3-Substituted ManNAc	34.81	0.60

^a^ Retention time of the derived alditol acetate derivative adjusted to that of 1,5-di-O-acetyl-2,3,4,6-tetra-O-methylglucitol-1-d. ^b^ Total ion count based on the detector response.

The high-resolution electrospray ionization mass spectrum results obtained were consistent with those of compositional and methylation analyses. We found the presence of the fragment ion at *m*/*z* 392.5 suggesting that HexNAc was attached to RhaOAc, and a fragment ion at *m*/*z* 554.5 was consistent with the consecutive addition of Hex (+162). Additional ions (*m*/*z* 757.5, 946.5 and 1108.5) corresponded to the consecutive addition of sugar residues HexNAc, RhaOAc and Hex, respectively. From these initial studies, we concluded that the *A. salmonicida* A450 LPS O-antigen seems to be identical to the one described for strains A449 and 80204 [12], which can be depicted as follows:

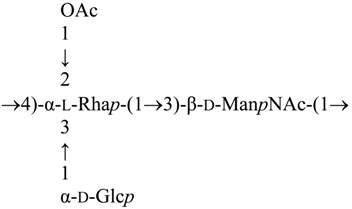



### 2.1. LPS O-Antigen Biosynthesis Gene Cluster (wb_salmo_)

**Figure 1:**
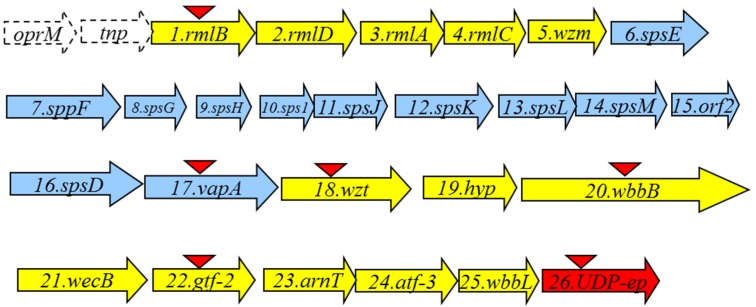
The *A. salmonicida* A450 LPS O-antigen (*wb*_salmo_) in yellow and S-layer cluster in blue open reading frames (ORFs) detected as complete genes. The genes, numbered according to the ORF number, were named according to the similarity found by their encoding products with proteins of well characterized functions. The *UDP-ep* gene (in red) does not belong to any of both clusters. The *orf* named *tnp* is encoding a putative transposase protein with a DDE domain from superfamily endonuclease. The *oprM* gene is incompletely sequenced and it should be noticed that it is also adjacent to the *wb* O34-antigen LPS from strain AH-3 previously characterized [16]. Triangles indicate the mutants obtained.

An *A. salmonicida* A450 cosmid-based genomic library was constructed and introduced into *E. coli* DH5α as previously described for other *Aeromonas* strains [15]. As we and others reported, *A. salmonicida* LPS O-antigen contains rhamnose. Thus, we used previously constructed DNA probes from *A. hydrophila* strain AH-3 *rmlA* and *B* genes (two biosynthetic rhamnose genes, [16]) due to their high DNA sequence conservation among *Aeromonas* strains, and screened the *A. salmonicida* A450 genomic library by colony Southern blot. Several tetracycline-resistant clones able to cross react with both probes were isolated and sequences flanking the DNA inserted were determined by using oligonucleotides complementary to the pLA2917 [15] cosmid. To complete the nucleotide sequence (GenBank KR704893) other sequence-derived oligonucleotides were designed by us, purchased (Sigma-Aldrich) and used. Analysis of the sequenced regions showed 26 complete putative open reading frames (ORFs) transcribed in the same direction, being 13 of them (ORF1 to 5 and ORF18 to 25) genes involved in the LPS O-antigen biosynthesis (*wb*_salmo_ cluster) as indicated in Figure 1 (in yellow).

The putative transposase protein showed a DDE domain from a superfamily of endonucleases. This domain contains three carboxylate residues that are believed to be responsible for coordinating metal ions needed for catalysis. The catalytic activity of this enzyme involves DNA cleavage at a specific site followed by a strand transfer reaction.

We observed several ORFs (6 to 17), indicated in blue (Figure 1), between the genes involved in the LPS biosynthesis that belong to a type II secretion system (T2SS) plus the structural gene *vapA* (ORF17) which encodes the surface A-layer protein. It seems logical that these ORFs are genes that encode for the production and export/assembly of the *A. salmonicida* A-layer characteristic from these strains. Interestingly, the insertion point of the A-layer genes is immediately downstream of a gene encoding for a Wzm putative protein. Downstream of the A-layer genes, a complete ORF encoding a Wzt putative protein was observed. Wzm and Wzt proteins are characteristics of an ABC-2 type transporter. Finally, we found a gene (ORF26), labeled in red (Figure 1), that encodes for a NAD-dependent dehydratase or UDP-sugar epimerase (named accordingly *UDP-ep*). This gene does not initially belong to the *wb*_salmo_ cluster, because its mutation does not abolish the LPS O-antigen (see next section). Sequence analysis of the *wb*_salmo_ gene cluster revealed a conserved JUMPstart sequence with the 8 bp *ops* (operon polarity suppressor) sequence (GGCGGTAG) 119 bp upstream of the ORF1. The *ops* sequence is recognized by the bacterial antiterminator RfaH, which can be recruited by the transcription elongation complex to reduce pausing and termination at intergenic sites of polycistronic operons, allowing RNA polymerase to finalise transcription of the distal genes in large operons [17,18].

Analysis of ORFs from *wb*_salmo_ and S-layer clusters with their predicted function based on their homology to proteins of known function is shown in Table 2.

**Table 2:** Characteristics of the *A. salmonicida* A450 O-antigen LPS (*wb*_salmo_) and A-layer cluster ORFs.

ORF	Protein Name	Protein Size (in Amino Acid Residues)	Predicted Function	Homologous Protein with Known Function	Percentage in Amino Acid Identity/Similarity
**Inserted S-layer protein cluster**					
18	Wzt	438	ABC transporter ATP binding protein	Wzt multispecies *Aeromonas*	100/100
19	Hyp	216	Hypothetical protein with domain Sulfotransferase	Sulfotransferase *Vibrio cholerae*	38/54
20	WbbB	1122	*N*-acetyl glucosaminyl transferase	WbbB *Klebsiella pneumonaie*	63/77
21	WecB	370	UDP-*N*-acetyl glucosamine 2-epimerase	WecB *Serratia marcescens*	100/100
22	Gtf-2	355	Glycosyl transferase	Glycosyl transferase family group 2 *Vibrio choleare*	78/91
23	ArnT	457	Hypothetical protein with ArnT (4-amino-4-deoxy-l-arabinose transferase) domain	Hypothetical protein multispecies *Aeromonas*	100/100
24	Atf-3	348	Acetyl transferase family 3	Acetyltransferase *Serratia marcescens*	45/65
25	WbbL	288	Rhamnosyl transferase	-Glucosyl transferase family 2 *A. veronii* -Rhamnosyl transferase *E. coli*	-100/100 -43/67
26	UDP-ep	318	NAD-dependent dehydratase or UDP-sugar epimerase	NAD-dependent dehydratase or UDP-sugar epimerase multispecies *Aeromonas*	100/100
**A-layer protein cluster**					
6	SpsE	552	S-layer secretion system protein E	Type II secretion system (T2SS) protein E *A. salmonicida*	100/100
7	SpsF	395	S-layer secretion system protein F	Type II secretion system(T2SS) protein F *A. salmonicida*	100/100
8	SpsG	143	S-layer secretion system protein G	Type II secretion system (T2SS) protein G *A. salmonicida*	97/99
9	SpsH	131	S-layer secretion system protein H	Type II secretion system (T2SS) protein H *A. salmonicida*	96/98
10	SpsI	132	S-layer secretion system protein I	Type II secretion system (T2SS )protein I *A. salmonicida*	99/100
11	SpsJ	235	S-layer secretion system protein J	Type II secretion system (T2SS) protein J *A. salmonicida*	94/98
12	SpsK	288	S-layer secretion system protein K	Type II secretion system (T2SS) protein K *A. salmonicida*	100/100
13	SpsL	371	S-layer secretion system protein L	Type II secretion system (T2SS) protein L *A. salmonicida*	94/95

### 2.2. Mutant Isolation and Characterization

**Figure 2:**
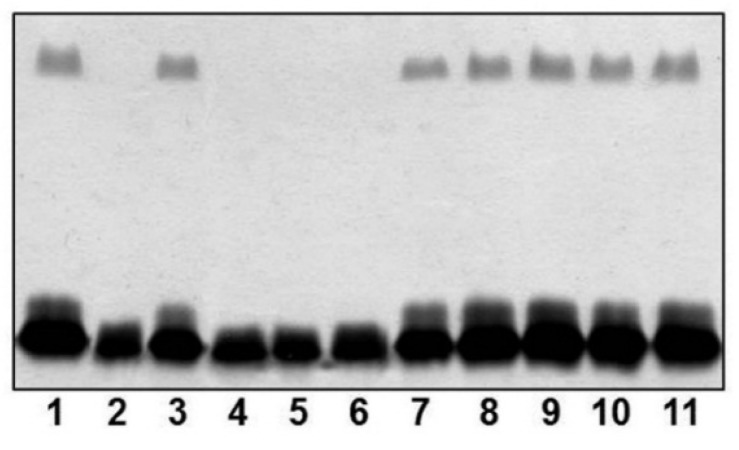
LPS analysed by SDS-PAGE (12%) and silver stained from *A. salmonicida* A450 (wild-type, lane 1), mutants A450ΔrmlB (lane 2), A450ΔvapA (lane 3), A450Δwzt (lane 4), A450ΔwbbB (lane 5), A450Δgtf-2, (lane 6), A450ΔUDP-ep (lane 7), mutant A450ΔrmlB complemented with A450*rmlB* (lane 8), mutant A450Δwzt complemented with A450*wzt* (lane 9), mutant A450ΔwbbB complemented with A450*wbbB* (lane 10), and mutant A450Δgtf-2 complemented with A450*gtf-2* (lane 11).

As described in Materials and Methods section, we obtained in-frame mutants in ORFs 1, 17, 18, 20, 22 and 26 named A450ΔrmlB, A450ΔvapA, A450Δwzt, A450ΔwbbB, A450Δgtf-2 and A450ΔUDP-ep, respectively. As shown in Figure 2, mutants A450ΔrmlB, A450Δwzt, A450ΔwbbB and A450Δgtf-2 were unable to produce LPS O-antigen when analyzed in a SDS-PAGE gel. However, A450ΔvapA and A450ΔUDP-ep mutants showed in the same gels an identical LPS profile as the wild-type strain with O-antigen ladder repetitions.

**Figure 3:**
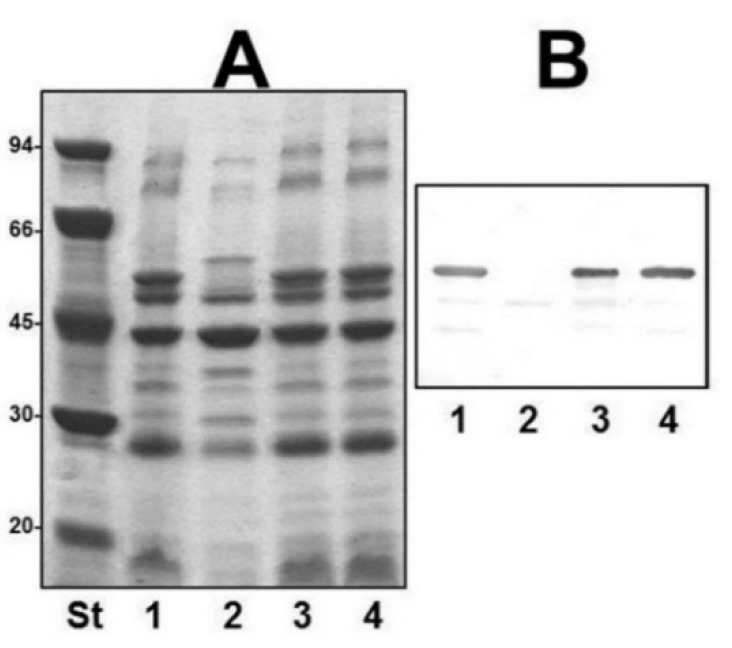
(**A**) Outer membrane proteins, and (**B**) Western blot using antiserum against A-layer protein (anti-VapA protein) of strains: A450 (wild type, lane 1), mutant A450ΔvapA (lane 2), A450ΔvapA complemented with A450 *vapA* (lane 3), and A450ΔUDP-ep (lane 4). St, molecular weight standard.

We obtained outer-membrane proteins (OMp) from A450ΔvapA mutant as described in Materials and Methods. Analysis by SDS-PAGE gels showed the lack of the major protein band of approximately 49 kDa compared with the wild type (Figure 3A). This band reacts with specific serum anti-VapA protein in Western blot analysis (Figure 3B). Mutant A450ΔUDP-ep showed no changes in OMp.

The reintroduction of the corresponding wild-type genes in A450ΔrmlB, A450Δwzt, A450ΔwbbB, and A450Δgtf-2 fully restored the LPS profile of the wild-type strain in silver-stained SDS gels (Figure 2). A similar situation was observed when wild-type *vapA* was reintroduced in mutant A450ΔvapA, the presence in the OMp profile of the 49 kDa protein reacting with specific serum anti-A protein was restored (Figure 3).

### 2.3. Different A. salmonicida Subspecies Strains

**Figure 4:**
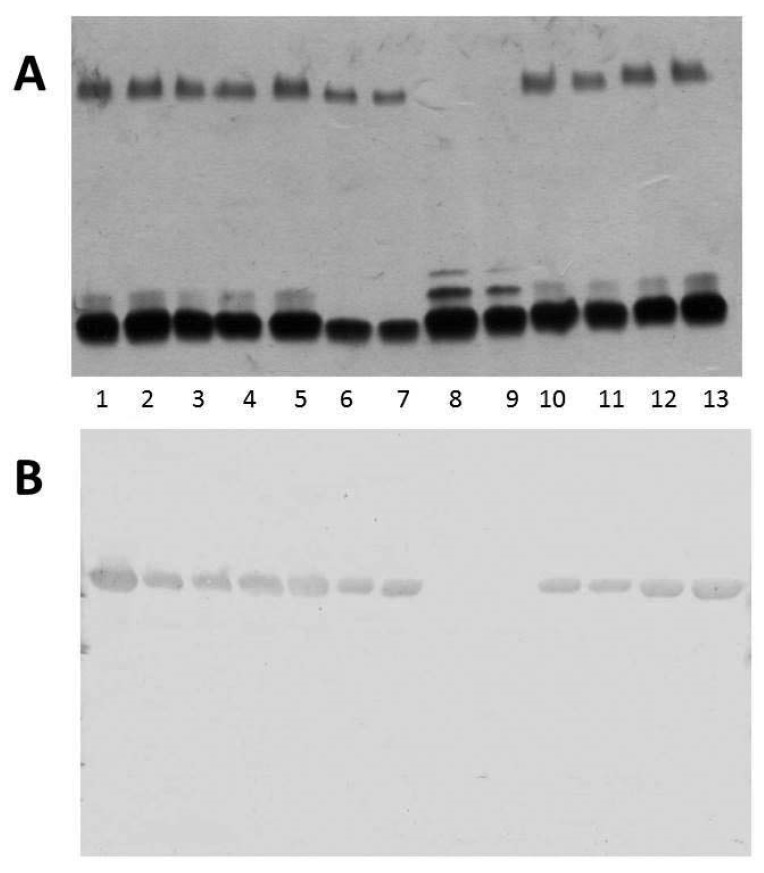
(**A**) LPS analysed by SDS-PAGE (12%) and silver stained from different *A. salmonicida* subspecies strains. Lane 1, subsp. *salmonicida* A450; lane 2, subsp. *masoucida* strain CECT896T; lane 3, subsp. *masoucida* strain AS60; lane 4, subsp. *achromogenes* strain CECT4238; lane 5, subsp. *achromogenes* strain CECT895T; lane 6, subsp. *achromogenes* strain AS46; lane 7, subsp. *achromogenes* strain AS102; lane 8, subsp. *pectinolytica* strain CECT5752T; lane 9, subsp. *pectinolytica* strain CECT5753; lane 10, subsp. *smithia* strain CECT5179; lane 11, subsp. *smithia* strain AS74; lane 12, subsp. *salmonicida* strain CECT894; and lane 13, subsp. *salmonicida* strain CECT4235; (**B**) Western blot analysis using specific serum anti-A protein and OMp from different *A. salmonicida* subspecies strains. Lanes as in A.

When we analyzed the LPS by SDS-PAGE gels from different *A. salmonicida* subsp. *salmonicida* strains, we found an identical O-antigen LPS profile in all of them. However, when we analyzed the LPS profile in gels from different *A. salmonicida* subspecies strains, we found that the strains belonging to *A. salmonicida* subsp. *pectinolytica* lack the characteristic LPS O-antigen homogeneous band, while the rest of the subspecies showed it (Figure 4A).

The analysis of the OMp from different *A. salmonicida* subspecies strains by Western blot using specific anti-A protein antiserum is shown in Figure 4B. With the exception of two strains identified as *A. salmonicida* subsp. *pectinolytica*, all other strains produced a band of approximately 49 kDa which reacted with antiserum against A-protein (Figure 4B).

### 2.4. Analysis of Fully Sequenced Genomes

**Figure 5:**
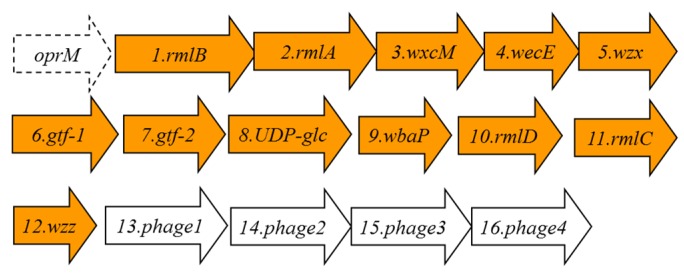
The *A. salmonicida* subsp. *pectinolytica* strain 34melT putative genes for the LPS O-antigen cluster in orange. The genes, numbered according to the ORF number, were named according to their similarity, as found by their encoding proteins, with proteins of well characterized functions.

When we inspected all the currently available *Aeromonas salmonicida* genomes, we found two for *A. salmonicida* subsp. *salmonicida* strains A449 and 01-B526, one for *A. salmonicida* subsp. *achromogenes* strain AS03, one for *A. salmonicida* subsp. *masoucida* strain NBRC 13784, one for *A. salmonicida* subsp. *pectinolytica* strain 34melT, and none for *A. salmonicida* subsp. *smithia*. Despite the different genome annotations (in part because these DNA regions had not been properly studied) with a more accurate comparative genomic analysis, we can conclude that in all the *A. salmonicida* genomes, besides the one for *A. salmonicida* subsp. *pectinolytica* strain 34melT, the *wb*_salmo_ is nearly identical. Furthermore, the chromosomal location between the *oprM* and *UDP-ep* genes is conserved among them. However, in the *A. salmonicida* subsp. *pectinolytica* strain 34melT genome, we found a putative *wb* cluster completely different. Briefly, the *rml* genes for rhamnose biosynthesis are not together, *wzm*-*wzt* are not present, and besides finding an *oprM* gene upstream, downstream of the *rmlC* seems to be a region with partial genes and encoded bacteriophage proteins (see Figure 5 and Table 3 ).

**Table 3:** Characteristics of the proteins encoded by the ORFs detected in their LPS O-antigen cluster.

ORF	Protein Name	Protein Size in Amino Acid Residues	Predicted Function
1	RmlB	361	dTDP-glucose-4-6-dehydratase RmlB
2	RmlA	289	Glucose-1-phosphate thymidylyl transferase RmlA
3	WxcM	137	dTDP-6-deoxy-3,4-keto-hexulose isomerase.
4	WecE	367	aminotransferase family, WecE
5	Wzx	416	O-antigen flippase
6	Gtf-1	140	glycosyl transferase group 1
7	Gtf-2	249	glycosyl transferase group 2
8	UDP-glc	388	UDP-glucose 6-dehydrogenase
9	WbaP	423	polyprenyl glycosyl phosphotransferase
10	RmlD	296	dTDP-4-dehydro rhamnose reductase
11	RmlC	176	dTDP-4-dehydro rhamnose 3,5-epimerase
12	Wzz	202	O-antigen size regulator protein
13	Phage1	113	Phage terminase 1 protein
14	Phage2	283	phage portal protein
15	Phage3	141	phage prohead peptidase
16	Phage4	398	putative phage phi-C31 gp36 major capsid-like protein

## 3. Discussion

The *A. salmonicida* A450 LPS O-antigen gene cluster (*wb*_salmo_) showed a G + C percentage of approximately 49.1%, which is significantly lower than the expected (59%–63%) for this species. This is characteristic of the *wb* clusters from different bacteria that can be 10% lower in G + C than the species average. Initially the encoded proteins showed some consistency with the chemical structure of the O-antigen LPS. ORF1 to 4 (in order RmlB, D, A and C, respectively) are the biosynthetic proteins for dTDP-rhamnose production, ORF21 (WecB) is the enzyme that converts UDP-GlcNAc into UDP-ManAc [19], and rhamnose, ManAc, and Glc are the monosaccharide components of the LPS O-antigen. There is no need for UDP-Glc specific genes. Three glycosyl transferases seem to be involved in the biosynthesis of the O-antigen repeating unit (ORF20, named WbbB; ORF22, named Gtf-2 and ORF25, named WbbL). WbbL is a presumptive rhamnosyl transferase, while the WbbB showed a HexNAc transferase domain (here probably acetyl-*N*-mannosamine), and Gtf-2 could be the transferase for the Glc incorporation to the LPS O-antigen repeating unit. There is also an acetyl transferase (ORF24 named Atf-3) which is in agreement with the acetylated rhamnose of the LPS O-antigen.

ORF5 and 18 were similar to an ABC-2 type transport system integral membrane and an ATP-binding protein, respectively (Table 2). The ORF5 protein hydrophobicity analysis and identification of the putative transmembrane domains [20] indicate that it is an integral membrane protein. The ORF18 protein contains the sequence GHNGAGKS (amino acid residues 57 to 64) which corresponds to the Walker box A, a motif present in ATP-binding proteins, as well as the ABC transporter family signature YSSGMYVRLAFAVQA (amino acid residues 162–175). Thus, ORF5 and 18 were named *wzm* and *wzt* respectively, despite the fact that they are usually found adjacent. Two main known pathways for O-antigen export have been established [9], the Wzy-dependent pathway for heteropolysaccharide structures and the ABC-2 transporter-dependent pathway mainly for homopolysaccharides or disaccharides. The presence of complete Wzm (ORF5) and Wzt (ORF18) showed that this LPS O-antigen belongs to the second pathway and represents the first example of an O-antigen LPS polysaccharide with three different monosaccharides in their repeating unit assembled by this pathway. Between the Wzm (ORF5) and Wzt (ORF18), we found the genes responsible for the biosynthesis and assembly of the S-layer (named A-layer), (Figure 1 and Table 2). The S-layer-encoding genes included a large group of T2SS genes plus some encoding hypothetical proteins related to this specific S-layer T2SS system (ORF6 to 16), and the last gene encoding the unique protein that forms the A-layer (*vapA*, ORF17) characteristic of *A. salmonicida* strains. The insertion point of this genomic cluster seems to be in the intergenic region between the genes encoding for a Wzm protein, located just upstream of the S-layer genes, and the gene encoding a Wzt protein (ORF18, as previously indicated). Accordingly to this, the A450ΔvapA in frame mutant showed identical LPS profile as the wild-type strain with the same O-antigen profile in SDS-PAGE gels, but lacked the VapA protein being then unable to produce the A-layer. Mutants A450ΔrmlB, A450Δwzt, A450ΔwbbB, and A450Δgtf-2, when analyzed in an SDS-PAGE gel, were unable to produce LPS O-antigen. However, the A450ΔUDP-ep mutant showed an identical LPS profile as the wild-type strain with the presence of O-antigen in the LPS. Then, we concluded that ORF22 (UDP-ep) does not belong to the *wb*_salmo_, because it seems not to be need for LPS O-antigen biosynthesis.

It is characteristic of the LPS O-antigen disaccharides assembled and exported thought an ABC-2 type transporter to contain a large protein (WbbB), like in *K. pneumoniae* O12 [21,22], that shows two clear domains. The first domain, in *K. pneumoniae* WbbB consisted of a condensation or capsule polysaccharide biosynthesis domain from amino acid residues 125 to 300; and a second domain with glycosyltransferase activity from GT1 family (625 to 825 amino acid residues) related to HexNAc transferases [21]. The *A. salmonicida* WbbB (ORF20) is larger than the one in *Klebsiella*, and showed three domains: capsule polysaccharide biosynthesis (130 to 290 amino acid residues), a glycosyltransferase activity from GT1family 1 (500 to 825 amino acid residues), and an additional glycosyltransferase activity domain from GT2 family (850 to 1050 amino acid residues). The fact there is an additional glycosyltransferase domain in the *A. salmonicida* WbbB seems to be in accordance with the O-antigen being a trisaccharide instead of a disaccharide.

At this moment, not much can be said about the hypothetical protein with a sulfotransferase domain (ORF 19, named Hyp), no such residue seems to be found in the chemical structure of the LPS O-antigen in these strains [10,12], and also about the hypothetical protein (ORF23 named ArnT) with a 4-amino-4-deoxy-l-arabinose transferase domain. However, it is true that in *Aeromonas* has been described the presence of a 4-amino-4-deoxy-l-arabinose in the lipid A [23]. Both genes encoding these proteins are clearly conserved among the different *A. salmonicida wb*_salmo_. The biosynthesis of O-antigens starts with the assembly of monomers on an undecaprenol phosphate (lipid carrier), before their incorporation into the LPS molecules, by enzymes that can be present or not in the *wb* gene cluster [9]. In *wb*_salmo_ we could not find such enzyme.

**Figure 6:**
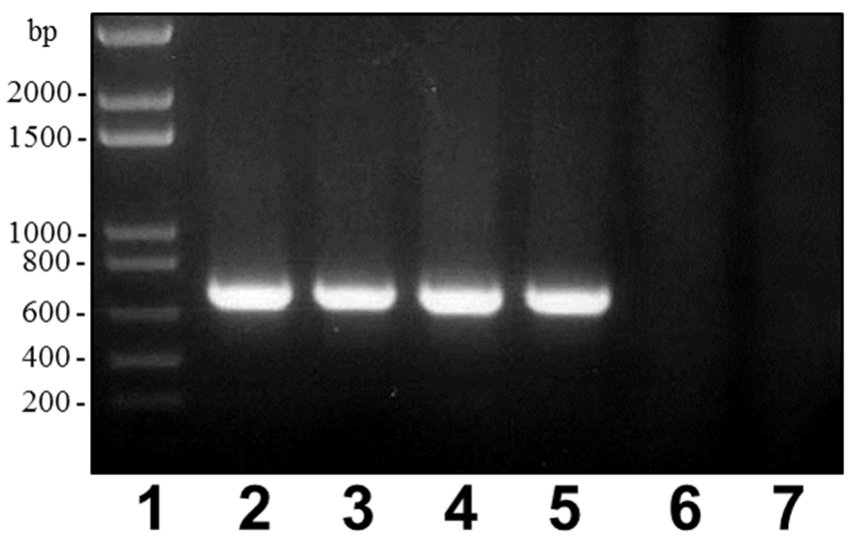
PCR amplified band (696 bp) with primers VapA-for: 5′-ATCGACAGCAATGGCAAG-3′ and VapA-rev: 5′-ATCACGGGTGAGGATGAAG-3′ and *A. salmonicida* genomic DNAs from strains: subspecies *salmonicida* A450 (lane 2), subspecies masoucida CECT4235 (lane 3), subspecies *achromogenes* CECT4238 (lane 4), subspecies *smithia* CECT5179 (lane 5), subspecies *pectinolytica* CECT5752T (lane 6), and subspecies *pectinolytica* CECT5753 (lane 7). Lane 1, DNA molecular weight standard.

When we inspected and deeply studied all the currently available *A. salmonicida* genomes, we found that the *wb*_salmo_ was conserved in all of the different subspecies with identical gene location besides in *A. salmonicida* subsp. *pectinolytica*. This fact is in full agreement with the LPS profiles in SDS-PAGE obtained for the different *A. salmonicida* strains from several subspecies. Sequence analysis of the *A. salmonicida* subsp. *pectinolytica* *wb* gene cluster from strain 34melT revealed that the LPS O-antigen seems to be exported through a Wzx-Wzy pathway and not an ABC-2 transporter-dependent pathway. However, besides the presence of the typical genes encoding the Wzx and Wzz proteins, no gene encoding a Wzy protein could be found in this cluster. Wzy mutants showed only a single O-antigen repeating unit [9] and, in the case of the LPS profile of *A. salmonicida* subsp. *pectinolytica* strains, it seems that a single O-antigen unit could be observed. *A. salmonicida* subsp. *pectinolytica* strains clearly show a different O-antigen LPS compared to the other subspecies and lack the A-layer according to our results. No such A-layer protein encoded by *vapA* gene could be detected by blot hybridization or PCR using appropriate DNA primers (Figure 6). This gene could not be found in the complete genome of *A. salmonicida* subsp *pectinolytica* strain 34melT.

These two features, lack of *wb*_salmo_ and A-layer, are in agreement with the fact that atypical *A. salmonicida* subsp. *pectinolytica* strains are the only ones not found as pathogens in a wide variety of fish species [24]. Besides, no genome is available for *A. salmonicida* subsp. *smithia.* Our results suggest that the *wb*_salmo_ should also be conserved in these strains, according to the LPS profile in SDS-PAGE gels.

## 4. Materials and Methods

### 4.1. Bacterial Strains, Plasmids and Growth Conditions

The bacterial strains and plasmids used in this study are listed in Table 4. *E. coli* strains were grown on Luria-Bertani (LB) Miller broth and LB Miller agar at 37 °C, while *Aeromonas salmonicida* strains were grown either in tryptic soy broth (TSB) or agar (TSA) at 20 °C. Ampicillin (100 µg·mL^−1^), chloramphenicol (25 µg·mL^−1^), tetracycline (20 µg·mL^−1^), kanamycin (50 µg·mL^−1^), nalidixic acid (20 µg·mL^−1^) and gentamycin (20 µg·mL^−1^) were added to the different media when required. *A. salmonicida* AS46, AS60, AS74, and AS102 were kindly provided by Prof. Brian Austin (University of Stirling, Scotland).

#### 4.1.1. General DNA Methods

General DNA manipulations were done essentially as previously described [30]. DNA restriction endonucleases, T4 DNA ligase, *E. coli* DNA polymerase (Klenow fragment), and alkaline phosphatase were used as recommended by the Sigma-Aldrich (St Louis, MO, USA).

#### 4.1.2. DNA Sequencing and Computer Analysis of Sequence Data

Double-stranded DNA sequencing was performed by using the dideoxy-chain termination method [31] from PCR amplified DNA fragments with the ABI Prism dye terminator cycle sequencing kit (PerkinElmer, Barcelona, Spain). Oligonucleotides used for genomic DNA amplifications and DNA sequencing were purchased from Sigma-Aldrich (St Louis, MO, USA). Deduced amino acid sequences were compared with those of DNA translated in all six frames from non-redundant GenBank and EMBL databases by using the BLAST [32] network service at the National Center for Biotechnology Information and the European Biotechnology Information. ClustalW was used for multiple-sequence alignments [33].

**Table 4:** Bacterial strains and plasmids used.

Strain or Plasmid	Relevant Characteristics	Reference or Source
***E. coli* strains**		
DH5α	F^−^ *end A hsdR17* (rK^−^ mK^+^) *supE44 thi-1 recA1 gyr-A96* _*80lacZ*M15	[25]
MC1061	*thi thr1 leu6 proA2 his4 argE2 lacY1 galK2 ara14 xyl5,* *supE44*, *λpir*	[26]
***A. salmonicida* strains**		
A450	Wild type, subsp. *salmonicida*	[27]
A450nal ^R^	A450 nalidixic acid resistant	[27]
A450ΔrmlB	A450 *rmlB* in frame mutant unable to produce LPS O-antigen	This study
A450Δwzt	A450 *wzt* in frame mutant unable to produce LPS O-antigen	This study
A450ΔwbbB	A450 *wbbB* in frame mutant unable to produce LPS O-antigen	This study
A450Δgtf-2	A450 *gtf-2* in frame mutant unable to produce LPS O-antigen	This study
A450ΔvapA	A450 *vapA* in frame mutant, unable to produce A-layer but able to produce LPS O-antigen	This study
A450ΔUDP-ep	A450 *UDP-ep* in frame mutant, able to produce LPS O-antigen and A-layer	This study
CECT894	Wild type, subsp. *salmonicida*	CECT
CECT4235	Wild type, subsp. *salmonicida*	CECT
CECT896T	Wild type, subsp. *masoucida*	CECT
AS60	Wild type, subsp. *masoucida*	[28]
CECT4238	Wild type, subsp. *achromogenes*	CECT
CECT895T	Wild type, subsp. *achromogenes*	CECT
AS46	Wild type, subsp. *achromogenes*	[28]
AS102	Wild type, subsp. *achromogenes*	[28]
CECT5752T	Wild type, subsp. *pectinolytica*	CECT
CECT5753	Wild type, subsp. *pectinolytica*	CECT
CECT5179	Wild type, subsp. *smithia*	CECT
AS74	Wild type, subsp. *smithia*	[28]
**Plasmids**		
pGEMT easy	PCR generated DNA fragment cloning vector Amp ^R^	Promega
pBAD33-Gm	Arabinose-inducible expression vector, Gm ^R^	[27,29]
pDM4	*pir* dependent with *sacAB* genes; oriR6K; Cm ^R^	[27]
pLA2917	Cosmid vector, Km ^R^, Tc ^R^	[15]

^R^ = resistant, CECT = SPANISH TYPE CULTURE COLLECTION.

#### 4.1.3. Mutant and Plasmid Constructions, Mutant Complementation Studies

The chromosomal in-frame A450ΔrmlB, A450ΔvapA, A450Δwzt, A450ΔwbbB, A450Δgtf-2 and A450ΔUDP-ep deletion mutants were constructed by allelic exchange as described by Milton *et al*. [26]. Plasmids were transferred to *A. salmonicida* strains as previously indicated [27]. To complete the allelic exchange, the integrated suicide plasmid was forced to recombine out of the chromosome by growing on agar plates containing 10% sucrose. Mutants were selected based on their survival on plates containing 10% sucrose and loss of the chloramphenicol resistant marker of vector pDM4. The mutations were confirmed by sequencing of the whole constructs in amplified PCR products. The primers used are shown in Table 5.

Table 5(**A**) Primers used in the construction of chromosomal in-frame deletion mutants. (**B**) Primers used for mutant complementation using vector pBAD33-Gm.

**Table:** 

A	
Primers ^a,b^	Amplified Fragment
*rmlB*	
A: 5′-CGCGGATCCCAAGTTCTGCCTGGTAT-3′	AB (632 bp)
B: 5′-*TGTTTAAGTTTAGTGGATGGG*TGCACCACCAGTGACAAG-3′	
C: 5′-*CCCATCCACTAAACTTAAACA*AGTGGTGCCTACCAATCCT-3′	CD (704 bp)
D: 5′-CGCGGATCCAACATCGGGTTTGCTCT-3′	
	AD (1312 bp)
*vapA*	
A: 5′-GAAGATCTGCCGATTCAGGTAAAACAG-3′	AB (717 bp)
B: 5′-*TGTTTAAGTTTAGTGGATGGG*GCTAATCACGACATCAGCA-3′	
C: 5′-*CCCATCCACTAAACTTAAACA* GAAGGCGTGGATATTCAGA-3′	CD (670 bp)
D: 5′-GAAGATCTAACGATCATCCATCTCTCG-3′	
	AD (1366 bp)
*wzt*	
A: 5′-CGCGGATCCGAGCTGGCTGATCTCTTCA-3′	AB (721 bp)
B: 5′-*TGTTTAAGTTTAGTGGATGGG*GGAACGATAGATGGGAAATG-3′	
C: 5′-*CCCATCCACTAAACTTAAACA*GATGTCGCCATGTTTCAAG-3′	CD (653 bp)
D: 5′-CGCGGATCCTGATTGGGCGAAAATA-3′	
	AD (1353 bp)
*wbbB*	
A: 5′-CGCGGATCCTACTTGCCCGAGATACCAG-3′	AB (659 bp)
B: 5′-*TGTTTAAGTTTAGTGGATGGG*ACCTAGCACGACCCAAAG-3′	
C: 5′-*CCCATCCACTAAACTTAAACA*GTTAAGCAGGCGCTATTTG-3′	CD (753 bp)
D: 5′-CGCGGATCCTACGATGCGATGTTACCAA-3′	
	AD (1391 bp)
*gtf-2*	
A: 5′-CGCGGATCCGCACCTACGCAAATTTCTC-3′	AB (722 bp)
B: 5′-*TGTTTAAGTTTAGTGGATGGG*CACCGGTGAAAGATAAACC-3′	
C: 5′-*CCCATCCACTAAACTTAAACA*TTTCATAATAGTGGCGATGC-3′	CD (631bp)
D: 5′-CGCGGATCCGACTGCCGTCTCTTTGAAC-3′	
	AD (1332 bp)
*UDP-ep*	
A: 5′-CGCGGATCCTGGCGTTGAATAATGGAG-3′	AB (646 bp)
B: 5′-*TGTTTAAGTTTAGTGGATGGG*CTTACCAACAAACCCGTTG-3′	
C: 5′-*CCCATCCACTAAACTTAAACA*AAGGCTCAGAGGCGATTAC-3′	CD (771 bp)
D: 5′-CGCGGATCCACCATCCCCCATAAAGAT-3′	
	AD (1395 bp)

**Table:** 

B		
Plasmid	Primers	Amplified Fragment
pBADGm-*rmlB* ^c^	RmlB-FOR: 5′-TCC**CCCGGG**TTAAAAGCAGCGAACTG-3′	1380 bp
	RmlB-REV: 5′-GCTCTAGACGCTGGAGTCAAAATCAAC-3′	
pBADGm-*vapA* ^c^	VapA-FOR: 5′-TCC**CCCGGG**TGATCAACGGATAGGTTCAA-3′	1666 bp
	VapA-REV: 5′-GCTCTAGAAGGGAACAAATGAAACTGCT-3′	
pBADGm-*wzt* ^c^	Wzt-FOR: 5′-TCC**CCCGGG**TGACCACAGCCCTTATTTC-3′	1473 bp
	Wzt-REV: 5′-GCTCTAGATGCAGTAGTCCCACCTTTT-3′	
pBADGm-*wbbB* ^d^	WbbB-FOR: 5′GGAATTCTAAGCTCACGGTTGCACAG-3′	3689 bp
	WbbB-REV: 5′-TCC**CCCGGG**ATAACCGGAGCCATTTTGAT-3′	
pBADGm-*gtf2* ^c^	gtf2-FOR: 5′-TCC**CCCGGG**ATGGCTAAAGGTTCTTCACC-3′	1269 bp
	gtf2-REV: 5′-GCTCTAGACATGACTGAAATACCCTGGA-3′	

^a^ Italic letters show overlapping regions. ^b^ Underlined letters show *Bam*HI or *Bgl*II restriction site. ^c^ Primers contain *Sma*I(bold) and *Xba*I(underlined), the PCR amplified product was ligated to *Sma*I-*Xba*I digested pBAD33-Gm. ^d^ Primers contain *Eco*RI (doubleunderlined) and *Sma*I(bold), the PCR amplified product was ligated to *Eco*RI-*Sma*I digested pBAD33-Gm.

For complementation studies, the *A. salmonicida* A450 *rmlB*, *vapA*, *wzt*, *wbbB*, and *gtf-2* genes were PCR amplified using appropriate oligonucleotides obtained from the sequenced clones and chromosomal A450 DNA as template, ligated to plasmid pGEMT (Promega), and transformed into *E. coli* DH5α. After checked, the corresponding genes were subcloned on plasmid pBAD33-Gm (Table 5) with an arabinose-inducible and glucose-repressible promoter [27,29]. Induction was obtained by adding l-arabinose to a final concentration of 0.2% (*w*/*v*). Plasmids were transferred to *A. salmonicida* strains as previously indicated [27].

#### 4.1.4. LPS Characterization and SDS-PAGE

LPS was obtained after proteinase K digestion of whole cells [34] for screening purposes. LPS samples were separated by sodium dodecyl sulfate-polyacrylamide gel electrophoresis (SDS-PAGE) and visualized by silver staining as previously described [34].

#### 4.1.5. LPS Isolation and O-Deacetylation

Cells (3 g dried weight) were digested with DNase, RNase (24 h, 3 mg each) and Proteinase K (36 h, 3 mg) in 25 mM Tris-HCl buffer pH 7.63 containing 2 mM CaCl_2_ (30 mL), the suspension was dialysed against distilled water and freeze-dried. Digested cells were extracted with aqueous 45% phenol at 68 °C [14], the extract was dialysed against tap water without separation of the layers, residual cells were removed by centrifugation, and the supernatant was freeze-dried to give lipopolysaccharide sample. An aliquot (150 mg) was degraded with 0.1 M sodium acetate buffer pH 4.2 for 4 h at 100 °C, the lipid precipitate was removed by centrifugation (13,000× *g*, 20 min), and high-molecular-mass O-polysaccharide (40 mg) was isolated from the supernatant by gel-permeation chromatography on a column (50 × 2.5 cm) of Sephadex G-50 Superfine in pyridinium acetate buffer (4 mL pyridine and 10 mL HOAc in 1 L water) using a Knauer differential refractometer (Knauer, Germany) for monitoring. The polysaccharide was deacetylated by heating with aqueous 12% ammonia (2 mL) for 3 h at 60 °C, ammonia was removed by stream of air, and the remaining solution was freeze-dried.

*Sugar analysis and Electrospray liquid chromatography mass spectrometry analysis.* For sugar analysis, a polysaccharide sample (1 mg) was hydrolyzed with 2 M CF_3_CO_2_H (100 °C, 4 h), the monosaccharides were conventionally converted into the alditol acetates [35] and analyzed by GLC on a Varian 3700 chromatograph (Varian Inc., Palo Alto, CA, USA) equipped with a fused-silica gel SPB-5 column using a temperature gradient from 150 °C (3 min) to 320 °C at 5° min^−1^. The absolute configurations of the monosaccharides were determined as described [36], using the same GLC conditions as in sugar analysis.

Mass spectrometry studies of purified O-polysaccharide were performed in the negative ion mode using a microTOF II instrument (Bruker Daltonics). A sample of the O-polysaccharide (~50 ng·µL^−1^) was dissolved in a 1:1 (*v*/*v*) water-acetonitrile mixture and sprayed at a flow rate of 3 µL·min^−1^. Capillary entrance voltage was set to 4.5 kV and exit voltage to −150 V; drying gas temperature was 180 °C.

#### 4.1.6. OM Protein and S-Layer Isolation and Characterization

Outer membranes (OM) were obtained by incubating membrane suspensions with 3% Sarkosyl in 20 mM TrisHCl buffer (pH 8.0) for 20 min at room temperature, as previously described [37]. Protein was analysed by SDS-PAGE and separated protein bands were visualized by Coomassie Brilliant blue staining as previously described [37]. Anti-purified-A-layer antiserum was obtained and assayed as previously described [38]. After SDS-PAGE, immunoblotting was carried out as previously described [39].
